# Diagnosis of Mondor's Disease in the Emergency Department with Bedside Ultrasound

**DOI:** 10.1155/2015/817960

**Published:** 2015-01-18

**Authors:** J. Michael O'Neal, Erik Castleberg, Vi Am Dinh

**Affiliations:** ^1^Department of Emergency Medicine, Loma Linda University, 11234 Anderson Street, A-108, Loma Linda, CA 92354, USA; ^2^Division of Critical Care, Department of Internal Medicine, Loma Linda University, 11234 Anderson Street, A-108, Loma Linda, CA 92354, USA

## Abstract

Mondor's disease is a rare condition characterized by a superficial thrombophlebitis that can occur in the thoracoabdominal and genital areas. Findings with ultrasound in penile Mondor's disease are readily measurable: a noncompressible penile vein without flow and absence of tears of the corpus cavernosum or tunica albuginea, hematoma, or evidence of fracture of the penis. We present a case of Mondor's disease, diagnosed with bedside ultrasound, in the emergency department. Ultrasonography is readily available within the emergency department, and we suggest its use in aiding diagnosis of genitourinary disorders such as Mondor's disease.

## 1. Introduction

Mondor's disease is a relatively uncommon disease, first described by Mondor in 1939 referring to the superficial thrombophlebitis in the thoracoabdominal wall [1]. Manifestations of the disease have subsequently been noted on the penis, groin, axilla, antecubital fossa, abdominal wall, and posterior cervical region [1].

The true incidence of Mondor's disease is unknown, but one series showed an incidence of 18 of 1296 (1.39%) patients in a sexually transmitted disease clinic over a 12-year period [2]. The study demonstrated an association with several sexual behaviors in the patients, including a history of vigorous sex after a period of abstinence in 17 of the 18 patients, which is consistent with the presentation of the patient presented here.

## 2. Case Presentation

A 24-year-old previously healthy male presented to the emergency department complaining of five days of painful penile swelling after experiencing a “popping” sensation during intercourse. The swelling was described as being at the base of the penis and extending down the shaft. The patient reported intermittent swelling of the penile shaft lasting between four and five days for the past three to four months. He described vigorous intercourse preceding these events. The patient otherwise denied trauma, dysuria, hematuria, difficulty with erection, multiple sexual partners, or attempted intercourse since the “popping” sensation was noted.

Upon presentation, the patient was normotensive (138/83) with a normal heart (100) and respiratory (18) rate and was afebrile (36.8°C). Physical examination revealed a well-developed male in no distress without palpable hernias. Genitourinary exam revealed an uncircumcised penis and a palpable cord on the right dorsal side of the penis. Mild tenderness of the penile shaft was noted and testicular exam revealed no swelling or pain on palpation.

**Figure 1:**
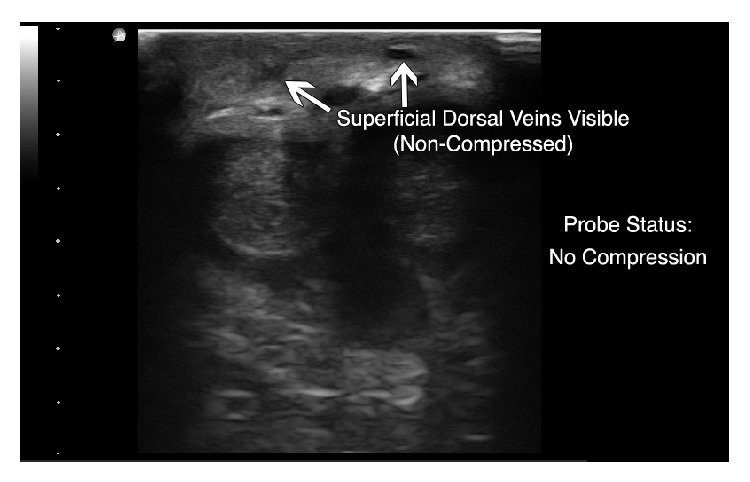
Noncompressed view of superficial dorsal veins with hypoechoic right sided vessel, suggesting superficial thrombophlebitis (depth set at 4 cm).

**Figure 2:**
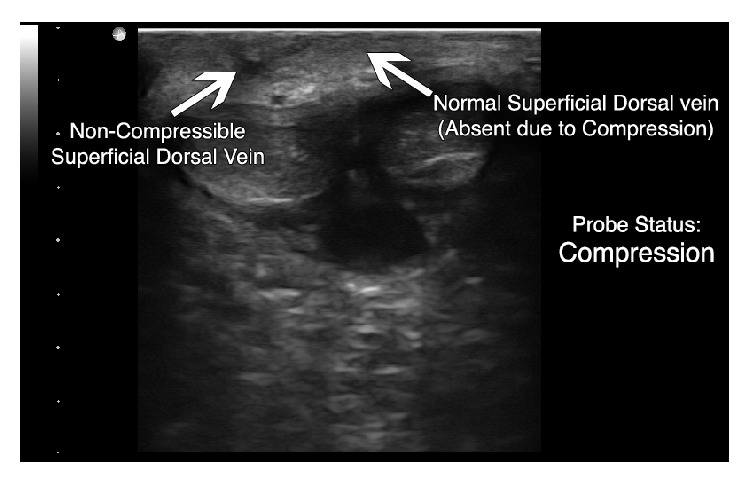
Compressed view of superficial dorsal veins showing noncompressible right superficial dorsal vein (depth set at 4 cm).

**Figure 3:**
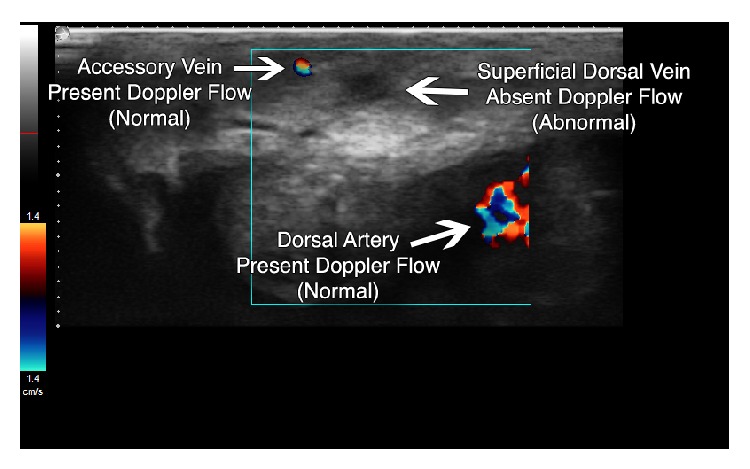
Color Doppler ultrasonography showing absent flow in right superficial vein when compared to accessory vein and dorsal artery, which both demonstrate normal flow (depth set at 2 cm).

Ultrasound of the penis using an Ultrasonix SonixTOUCH (Vancouver, British Columbia) machine, with a high frequency linear probe (L15-5) on the small parts setting, demonstrated a noncompressible, hypoechoic right lateral superficial dorsal vein (Figures 1 and 2). The uncompressed vein did not exhibit a reduction in caliber compared to additional superficial dorsal vein identified. Color Doppler ultrasonography demonstrated a lack of flow compared to the deep dorsal vein and dorsal arteries (Figure 3). The arteries and soft tissues of the penis were otherwise unremarkable.

Urology was consulted, and the patient was diagnosed with superficial thrombophlebitis (Mondor's disease) of the right lateral superficial dorsal vein without evidence of penile fracture. Conservative management with NSAIDs was recommended with outpatient follow-up to ensure the resolution of symptoms, which was expected to take up to 4 weeks. The patient was contacted by phone 8 weeks later and reports his symptoms had resolved within 4 weeks with ibuprofen treatment and cessation of intercourse.

## 3. Discussion

Ultrasound has been shown to have consistent features in Mondor's disease, including noncompressible veins and lack of venous color Doppler flow [1, 3, 4]. Other signs of thrombus include vein lumen size and thrombus echogenicity where a chronic thrombus has a smaller lumen size and increased echogenicity (i.e., hyperechoic) [5]. In this particular case, the vessel was noncompressible, lacked venous color Doppler flow, had a normal size lumen, and had decreased echogenicity (i.e., hypoechoic). These constellations of findings are consistent with an acute phase of Mondor's disease. These described findings are also found with Mondor's disease in other areas, such as the breast [1, 6]. Hye et al. additionally found weak flow and high resistance in nearby arteries using pulsed Doppler in examining Mondor's disease of the penis [7].

Mondor's disease can be diagnosed clinically with the findings of a palpable cord in the affected area without other significant findings beyond swelling [1, 3]. However, the disease is rare enough that many emergency providers may have some hesitancy in diagnosing the condition without supporting diagnostic evidence given that penile fracture is also an emergent condition in the differential for penile trauma. Kervancioglu et al. demonstrated the findings that would support the diagnosis of penile fracture on ultrasound, such as tears of the corpus cavernosum and tunica albuginea, as well as the presence of hematomas. Vascular injuries to the superficial dorsal vein, deep dorsal vein, dorsal artery, and deep cavernous artery may also be seen sonographically in penile fractures [8]. The absence of these additional sonographic findings can be used by the emergency providers to support the benign diagnosis, such as in this case.

Further imaging studies have been used for the diagnosis of superficial thrombophlebitis. For the cases of the disease occurring in the breast, mammography is commonly used, showing densities along the affected area [1, 9]. MRI angiography can also demonstrate thrombus and be used to evaluate extension of the thrombus, even into areas difficult or impossible to image with ultrasound or presence of hematoma; however, MRI is expensive and adds little to the clinical management of the disease [10]. Belleflamme et al. have suggested ultrasound be the confirmatory imaging modality of choice [11].

A case series by Al-Mwalad et al. [3] covered 25 patients over a six-year period with Mondor's disease of the penis with symptoms of feeling of tension in various locations of the penis without pain [3]. Improvement with conservative treatment was shown in 23 of 25 patients, with the remainder requiring surgical intervention, demonstrating that Mondor's disease of the penis is a relatively benign condition. Causative factors for Mondor's disease have not been definitively identified, though trauma, tumors, and surgery are considered risk factors [3]. One case study suggested urogenital infection and muscular strain as possible causes [12]. Immunohistochemistry demonstrated thrombophlebitis as the underlying pathology in most cases of Mondor's disease with occasional lymphangitis as a cause [13].

Proper identification of Mondor's disease assisted by ultrasonography allows for proper management of the disease. Patients diagnosed in the emergency department should be given proper follow-up, which may include testing for protein C and protein S or antithrombin III deficiencies [3], evaluation for other thrombophilic conditions [11], and possible search for occult malignancy [1, 11]. Treatment in the interim should consist of NSAIDs and cessation of intercourse [1, 3].

## 4. Conclusion

Ultrasound has been shown to be an effective means of supporting the diagnosis of Mondor's disease of the penis. Findings on ultrasound are a noncompressible penile vein without flow and absence of tears of the corpus cavernosum or tunica albuginea, hematoma, or evidence of fracture of the penis. Its use has been validated and accepted by specialties outside of the emergency department. Given the availability and low cost of the modality within the ED, familiarity with the sonographic findings can lead to the fact that emergency providers are able to quickly diagnose this rare condition at bedside with relative certainty.
